# Melinjo (*Gnetum gnemon* L.) Seed Extract Decreases Serum Uric Acid Levels in Nonobese Japanese Males: A Randomized Controlled Study

**DOI:** 10.1155/2013/589169

**Published:** 2013-12-17

**Authors:** Hiroyuki Konno, Yoshiaki Kanai, Mikiyuki Katagiri, Tami Watanabe, Akemi Mori, Tomoki Ikuta, Hiroko Tani, Shinobu Fukushima, Tomoki Tatefuji, Takuji Shirasawa

**Affiliations:** ^1^Department of Aging Control Medicine, Juntendo University Graduate School of Medicine, Bunkyo-Ku, Tokyo 113-0033, Japan; ^2^Institute for Bee Products & Health Science, Yamada Bee Company, Inc., 194 Ichiba, Kagamino-cho, Okayama 708-0393, Japan

## Abstract

Melinjo (*Gnetum gnemon* L.) seed extract (MSE) containing *trans*-resveratrol (3,5,4′-trihydroxy-*trans*-stilbene) and other derivatives exerts various beneficial effects. However, its mechanism of action in humans remains unknown. In this study, we aimed to investigate beneficial effects of MSE in healthy adult males. In this double-blind, randomized controlled study, 30 males aged 35–70 years with ≤10% flow-mediated dilatation received placebo or 750 mg MSE powder for 8 weeks, and twenty-nine males (45.1 ± 8.8 years old) completed the trial. There was a significant difference in the melinjo and placebo groups. Compared with the placebo control, MSE significantly reduced serum uric acid at 4 weeks and 8 weeks (*n* = 14 and 15, resp.). HDL cholesterol was significantly increased in the melinjo group. To clarify the mechanism of MSE for reducing uric acid, we investigated xanthine oxidase inhibitory activity, angiotensin II type 1 (AT1) receptor binding inhibition rate, and agonistic activities for PPAR**α** and PPAR**γ**. MSE, *trans*-resveratrol, and a resveratrol dimer, gnetin C (GC), significantly inhibit AT1 receptor binding and exhibit mild agonistic activities for PPAR**α** and PPAR**γ**. In conclusion, MSE may decrease serum uric acid regardless of insulin resistance and may improve lipid metabolism by increasing HDL cholesterol.

## 1. Introduction

Melinjo (*Gnetum gnemon* L.) belongs to the family Gnetaceae, native to Indonesia. The tree is small to medium in size, 15–20 m tall, with evergreen leaves. The fruit-like strobilus consists of little skin and a large nut-like seed that is 2–4 cm long inside, with both the fruits and leaves being very popular in Indonesian cuisines.

Kato et al. found that melinjo seed extract (MSE) contains various stilbenoids including *trans*-resveratrol (3,5,4′-trihydroxy-*trans*-stilbene), gnetin C (GC; resveratrol dimer), gnetin L (GC derivative), gnemonoside A (GC-diglucoside), gnemonoside C (GC-monoglucoside), and gnemonoside D (GC-monoglucoside) [1]. These derivatives are collectively referred to as “Melinjo resveratrol.” Recently, *trans*-resveratrol has attracted considerable attention because it extended the lifespan of mice that were fed a high-calorie diet [2]. Moreover, human studies indicated that *trans*-resveratrol is beneficial in the management of diabetes [3] and cardiovascular diseases [4]. However, several *in vitro* studies on the resveratrol derivatives in MSE revealed its nutraceutical effects such as the inhibition of lipase and amylase, antibacterial properties [1], inhibition of angiogenesis [5], and immunostimulatory effects [6]. In mice, MSE was reported to suppress body weight gain and improve insulin resistance [7]. However, the clinical efficacy of MSE remains unknown in humans. Therefore, to evaluate the effects of MSE on humans, we designed this clinical study using healthy volunteers and evaluated the various biomarkers in association with metabolic syndrome.

## 2. Materials and Methods

### 2.1. Clinical Study Design

The present study was a randomized, double-blinded, and placebo-controlled trial with parallel groups. We conducted the study according to the guidelines in the Declaration of Helsinki. All procedures involving human participants were approved by the Shirasawa Clinical Research Center Ethical Review Board. Each participant provided written and informed consent prior to participation.

### 2.2. Participants

Adult males aged 35–70 years with ≤10% flow-mediated dilation (FMD), determined at the time of screening, were recruited for this study from August to September, 2011, through newspaper advertisements at the Shirasawa Clinical Research Center, Tatebayashi city, Gunma prefecture, Japan. Exclusion criteria included the following conditions: consumption of functional foods related to lipid and glucose metabolism; allergy to melinjo; drinking red wine that contains *trans*-resveratrol regularly; receiving medication for hypertension, diabetes, or hyperlipidemia; and preexisting severe liver, renal, or heart disease. In addition, we excluded the volunteers who participated in another clinical study within two months and were not judged to meet the conditions by the doctor responsible for the study.

The enrolled participants who met the inclusion criteria were randomly assigned into the melinjo and placebo groups by using computer-generated random numbers. Each participant in the melinjo group consumed five capsules containing 750 mg MSE powder every morning (once daily) for eight weeks, while each participant in the placebo group consumed five placebo capsules following the same protocol. The participants could not distinguish the difference between the two types of capsules with respect to their shape, size, weight, and color. The participants were advised not to consume other health foods during the study. They attended the Shirasawa Clinical Research Center for clinical assessment at the following three study time points: baseline (0), 4, and 8 weeks. Finally, 29 adult males (age: 45.1 ± 8.8 years, BMI: 24.4 ± 1.9 kg/m^2^) participated in and completed the trial.

### 2.3. Test Substances

All MSE and placebo capsules were supplied by the Yamada Bee Company, Inc. The seeds (endosperms) of melinjo were collected in Indonesia (Desa Bangkok, Kecamatan Gurah Kabupaten Kediri, Kediri, Jawa Timur) in July 2009. The dried endosperms of melinjo (250 g) were powdered and soaked in 55% EtOH (750 mL) at room temperature for 3 days to obtain MSE (23 g). Dextrin (0.39 g) and water (5 g) were added to 6.25 g MSE and lyophilized to prepare the MSE powder used for biological experiments. In order to confirm the safety of the MSE powder, the Yamada Bee Company, Inc. conducted a repeated dose study in humans. Every morning for 28 days, 44 healthy volunteers aged 32–49 years were administered a maximum of 5,000 mg MSE powder. Throughout the study, no clinically noteworthy abnormalities were observed (unpublished data).

In our study, one melinjo capsule contained 150 mg MSE powder, 100 mg dextrin, 29 mg cellulose, and 9 mg sugar ester. The MSE powder contained >20% the resveratrol derivatives. Regarding *trans*-resveratrol, the content ratio was 0.1%. On the other hand, one placebo capsule contained 250 mg dextrin, 29 mg cellulose, and 9 mg sugar ester. The appearance of the capsules used for both groups was identical.

### 2.4. Clinical Assessments

The participants were instructed to arrive without having consumed anything for at least 8 h on the examination day. Before the initiation of the trial (week 0) and again at four and eight weeks, all participants went through FMD, pulse wave velocity (PWV), ankle-brachial index (ABI), body weight, fat percentage, BMI, and a general examination including blood pressure, pulse rate, and blood chemistry analysis (levels of total protein, albumin, albumin globulin ratio, total bilirubin, aspartate aminotransferase, alanine transaminase, *γ*-GTP, total cholesterol, HDL cholesterol, low-density lipoprotein (LDL) cholesterol, arteriosclerotic index, remnant-like particles (RLP) cholesterol, triglycerides, uric acid, urea nitrogen, creatinine, sodium, potassium, chlorine, HOMA-IR, fasting immunoreactive insulin, blood sugar, hemoglobin A1c, total homocycteine, and N-terminal prohormone of brain natriuretic peptide (NT-proBNP); the reactive oxygen metabolites-derived compounds (d-ROMs) test; the Biological Antioxidant Potential (BAP) test; white blood cell and red blood cell counts; hemoglobin levels; hematocrit value; mean cell volume; levels of mean cell hemoglobin, mean cell hemoglobin concentration, and platelets), and urinalysis (specific gravity, pH, protein, glucose, ketones, blood, bilirubin, and urobilinogen) (Table 1).

For the evaluation of endothelial function in metabolic syndrome, FMD was measured in the right brachial artery using UNEXEF38G (UNEX Corporation, Nagoya, Japan); specialized in measuring FMD, this device is a combination of ultrasonography and a sphygmomanometer. The probe of this device is composed of two probes to capture the minor axis of the vessel and one probe to capture the long axis of the vessels during the two probes. With the three probes, the position of a brachial artery and the long axis can be easily located, and the vessel diameter can be accurately measured. In addition, the equipment can automatically measure FMD after 5 min of avascularization. The participants were reclined on the bed in a supine position during the FMD test. They were fitted with a cuff, which was positioned on the right upper arm, abutting the cubital fossa. We maintained the laboratory room temperature at 25 ± 1°C, taking into account its effects on the examination [8].

The arteriosclerosis index was assessed using PWV and ABI by BP-203RPE III (Omron Healthcare Co., Ltd, Tokyo, Japan). This device has four cuffs that can simultaneously measure blood pressure levels in both arms and both legs and automatically calculate ABI. Moreover, the device can record pulse waves via sensors in the cuffs, calculate the transmission distance from the right arm to each ankle according to body height, and automatically compute and output the bilateral brachial-ankle PWV (baPWV) values using the transmission time and distance.

### 2.5. Statistical Analysis

Statistical analyses were performed using SPSS version 20 (IBM Corporation, NY, USA). Statistical comparisons between groups were calculated using two-way factorial ANOVA. The factors were the assignment and the survey period, and the dependent variables were the evaluation criteria. Multiple comparisons were performed using Tukey's HSD test. Values of *P* < 0.05 were considered significant. Results are presented as means ± SD.

### 2.6. *In Vitro *Experiments

#### 2.6.1. Assay of Xanthine Oxidase Activity

The assay mixture consisting of 50 mL test solution and 50 mL enzyme solution (0.05 units/mL in 70 mM phosphate buffer, pH 7.5) was prepared immediately before use. After preincubation at 25°C for 15 min, the reaction was initiated by the addition of 100 mL substrate solution (800 mM xanthine in the same buffer). The assay mixture was incubated at 25°C for 30 min. The reaction was stopped by adding 20 mL of 1 M HCl and 20 mL volumes of diluted 20 mM potassium dihydrogenphosphate onto a Sunniest RP-AQUQ column (4.6 mm I.D. × 150 mm). The mobile phase was acetonitrile/20 mM potassium dihydrogenphosphate (1 : 99 v/v) at a flow rate of 0.8 mL/min. Further, uric acid was detected by its UV absorbance at 290 nm. The retention time of uric acid on this system was 4.9 min. A blank was prepared in the same way, but the enzyme solution was added to the assay mixture after adding 1 M HCl. One unit of XO is defined as the amount of enzyme required to produce 1 mmol of uric acid/min at 25°C. The XO inhibitory activity was expressed as the percentage inhibition of XO in the above assay system, calculated as (1 − *B*/*A*) × 100, where *A* and *B* are the activities of the enzyme without and with test material, respectively. The IC_50_ values were calculated from the mean values of data from the four determinations. The extracts were dissolved initially in EtOH, followed by dilution with the buffer; the final EtOH concentration was <5%. Allopurinol, a known XO inhibitor, was used as a positive control.

#### 2.6.2. Evaluation of the Binding Inhibition Rate of Angiotensin II Type 1 Receptor

Evaluation of the affinity of compounds for the human angiotensin II type 1 (AT1) receptor in the transfected HEK-293 cells was determined using a radioligand binding assay. The cell membrane homogenates (8 *μ*g protein) were incubated for 120 min at 37°C with 0.05 nM [^125^I][Sar^1^-Ile8] angiotensin II, both in the absence or presence of the test compound, in a buffer containing 50 mM Tris-HCl (pH 7.4), 5 mM MgCl_2_, 1 mM EDTA, and 0.1% BSA. Nonspecific binding was determined in the presence of 10 *μ*M angiotensin II. Following incubation, the samples were rapidly filtered under vacuum through glass fiber filters (GF/B, Packard) presoaked with 0.3% PEI and rinsed several times with ice-cold 50 mM Tris-HCl using a 96-sample cell harvester (Unifilter, Packard). Thereafter, the filters were dried and measured for radioactivity in a scintillation counter (Topcount, Packard) using a scintillation cocktail (Microscint 0, Packard). The results were expressed as a percent inhibition of the control radioligand specific binding. The standard reference compound was saralasin, which was tested in each experiment at several concentrations to obtain a competition curve from which its IC_50_ was calculated.

#### 2.6.3. Assay of Peroxisome Proliferator-Activated Receptor (PPAR) *α* and *γ* Agonist Activity

The COS-1 cells were collected by processing of trypsin, centrifuged at 1000 rpm for 3 min at 4°C. After removing the supernatant, the cells were seeded in 60 mm culture dishes at a density of 5 × 10^5^ cell/well in a 2 mL medium and cultured for 24 h at 37°C with the presence of 5% CO_2_. The Effectene Transfection Reagent (QIAGEN, Tokyo, Japan) was used to transform the cells. 150 *μ*L Buffer EC, 0.25 *μ*g pPPAR*α*-Gal4 (or pPPAR*γ*-Gal4), 1 *μ*g pGal4-Luc, 1 *μ*g pSEAP-control vector, and 18 *μ*L Enhancer were added into a 1.5 mL tube, and the contents in the tube were stirred with the vortex for 10 s. Subsequently, after leaving for 3 min at 25°C, 25 *μ*L Effectene was added to the tube. The contents were stirred with the vortex for 10 s and were left for 7 min at 25°C. The medium of the 60 mm culture dish was removed, and 4 mL fresh medium was introduced there during this time. Subsequently, 7 min later, 1 mL culture medium was added to the 1.5 mL tube and was suspended with a pipette. All the contents were dripped to the 60 mm culture dish, and the contents were incubated for 16 h at 37°C with the presence of 5% CO_2_.

The transformed cells were collected by processing trypsin. The cells were centrifuged at 1000 rpm for 3 min at 4°C, and the supernatant was removed. The cells were suspended in 10 mL culture medium and were seeded in a 96-well plate with 125 *μ*L medium for each well. The cells were then cultured for 1-2 h at 37°C with the presence of 5% CO_2_. The test samples (1.25 *μ*L) were added to each well and were cultured with gentle stirring for 24 h at 37°C in the presence of 5% CO_2_.

The medium (25 *μ*L) was removed from each well of the 96-well plate and was transferred to each well of a 96-well white plate. Thereafter, a solution for measurement of the luciferase activity was fused at 37°C, and the 100 *μ*L solution was added to the 100 *μ*L medium of the rest in each well. Each luminescence activity was measured after reacting for 35 min in a dark place. 25 *μ*L 1 × dilution buffer was added to each 25 *μ*L medium, which was collected from the 96-well plate. It was stirred gently and left for 30 min at 65°C. Thereafter, they were cooled down to 4°C and then back to 25°C. 90 *μ*L assay buffer was added to each well, was gently stirred, and was left for 5 min at 25°C. 10 *μ*L/MUP solution was added to each medium, and it was gently stirred. After reacting for 60 min at 25°C in a dark place, the fluorescence intensity (Ex = 360 nm, Em = 460 nm) based on 4-methylumbelliferone was measured.

## 3. Results

### 3.1. MSE Decreases the Serum Uric Acid Levels in Healthy Volunteers

In order to study the beneficial effects of MSE, we designed a clinical trial of MSE with healthy volunteers, wherein we evaluated the various biomarkers, including blood chemistry, CBCs, body weight, blood pressure, urinalysis, pulse wave velocity (PWV), flow-mediated dilatation (FMD), and HOMA-IR (a biomarker of insulin sensitivity) (Table 1).

**Figure 1:**
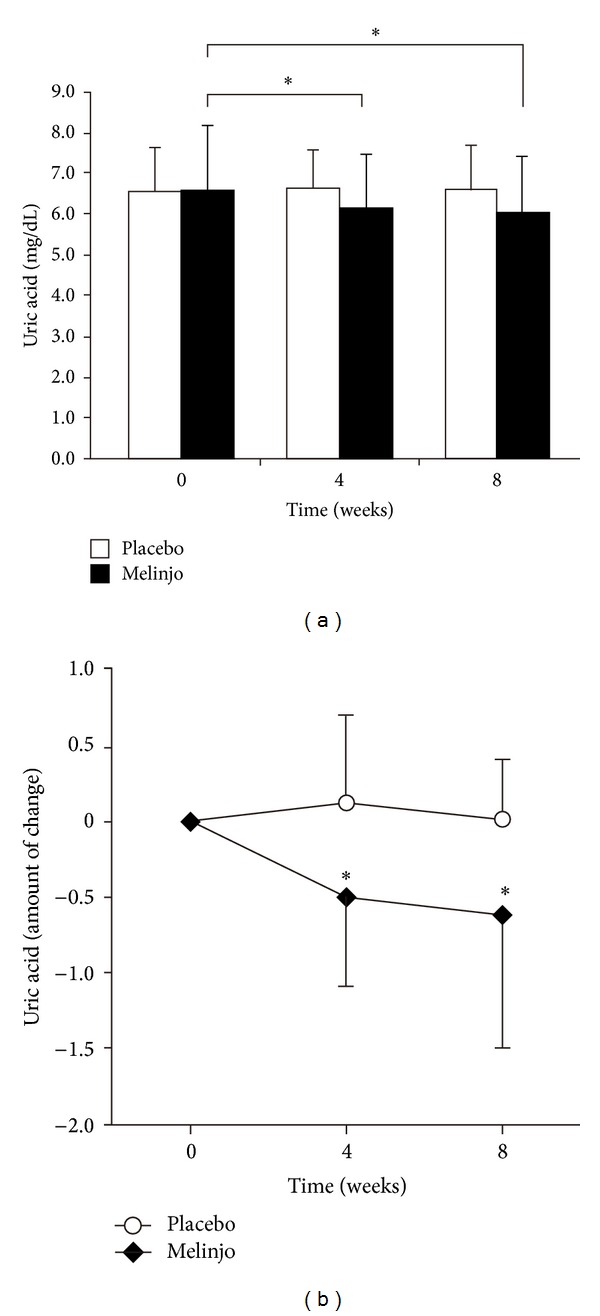
The effects of MSE on the serum uric acid levels before and four or eight weeks after the administration of 750 mg MSE or placebo. (a) The serum uric acid levels significantly decreased in the melinjo group (*n* = 14) than in the placebo group (*n* = 15). (b) The changes in the uric acid levels in the melinjo group were presented by an amount of changes in placebo group. The effect of MSE at four weeks was as well maintained at eight weeks. Statistical significance was calculated using Tukey's HSD test. Values are presented as means ± SD. *P < 0.05.

Healthy volunteers with administration of 750 mg MSE powder revealed a significant decrease in the uric acid levels at four weeks (6.3 ± 1.4 versus 6.7 ± 0.9 mg/dL, *P* < 0.05; Figure 1(a)) and at eight weeks (6.1 ± 1.4 versus 6.6 ± 1.1 mg/dL, *P* < 0.05; Figure 1(a)) when compared to the placebo control. As presented in Figure 1(b), we confirmed a beneficial effect of MSE at four weeks, which was as well maintained at eight weeks, suggesting the stable clinical benefit of MSE in the long-term control of serum uric acid levels.

Although a previous clinical study on *trans*-resveratrol demonstrated the improvements in insulin resistance and lipid profiles with human subjects [9], we failed to demonstrate these clinical benefits for MSE. Interestingly, in this study, we found a novel clinical benefit of MSE on uric acid. Because uric acid not only plays an important role as an antioxidant molecule but also as a biomarker for cardiovascular diseases and gout, we explored the possibility that MSE confers health benefits in the prevention of cardiovascular diseases and gout.

### 3.2. MSE May Inhibit AT1 Receptor Binding

**Figure 2:**
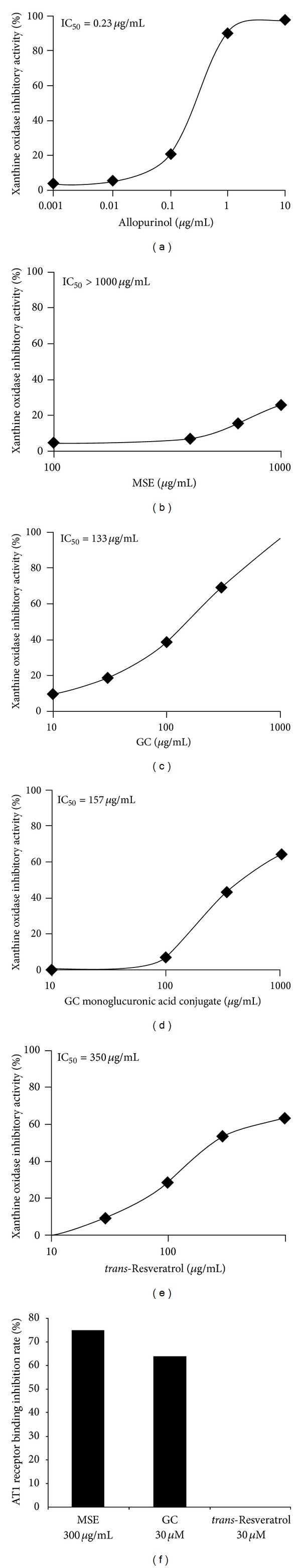
The effects of the 50% inhibitory concentration (IC_50_) of MSE, GC, GC monoglucuronic acid conjugate, and *trans*-resveratrol on the xanthine oxidase inhibitory activity. Allopurinol was used as a positive control. (a) Allopurinol. (b) MSE. (c) GC. (d) GC monoglucuronic acid conjugate. (e) *trans*-Resveratrol. (f) AT1 receptor binding inhibition rate of MSE, GC, and *trans*-resveratrol.

In order to clarify the mechanism of MSE in decreasing the serum uric acid levels, we investigated the potential inhibition of uric acid synthesis and uric acid reabsorption in the renal tubular epithelia. With regard to the synthesis of uric acid, we first investigated the inhibitory activity of MSE on xanthine oxidase. Allopurinol, a well-known chemical compound used for the treatment of gout, effectively inhibited the xanthine oxidase activity with IC_50_ at a concentration of 0.23 *μ*g/mL (Figure 2(a)). However, MSE, GC, GC monoglucuronic acid conjugate, and *trans*-resveratrol failed to demonstrate any inhibitory activities on xanthine oxidase (Figures 2(b)–2(e), IC_50_ at the concentrations of 133 *μ*g/mL, 157 *μ*g/mL, and 350 *μ*g/mL, resp.), suggesting that MSE decreases the serum uric acid levels by a mechanism other than the xanthine oxidase suppression. Next, we explored the possibility whether MSE inhibits the reabsorption of uric acid in the renal tubules. The inhibition of angiotensin decreases the serum uric acid levels by suppressing the reabsorption of uric acid from the renal tubular epithelia [10]. In this paper, we performed *in vitro* investigation to evaluate the inhibitory activity of MSE on angiotensin as well as GC, which revealed that MSE and GC have a significant inhibitory activity on AT1 receptor binding, whereas *trans*-resveratrol revealed no inhibitory activity. These data suggest that MSE inhibits the angiotensin signal, which then downregulates the transporter of uric acid; however, we cannot rule out the possibility that other pathways are involved in the regulation of uric acid because we have not evaluated all the relevant regulatory pathways.

### 3.3. MSE May Increase the Serum HDL Cholesterol Levels in Healthy Volunteers

**Figure 3:**
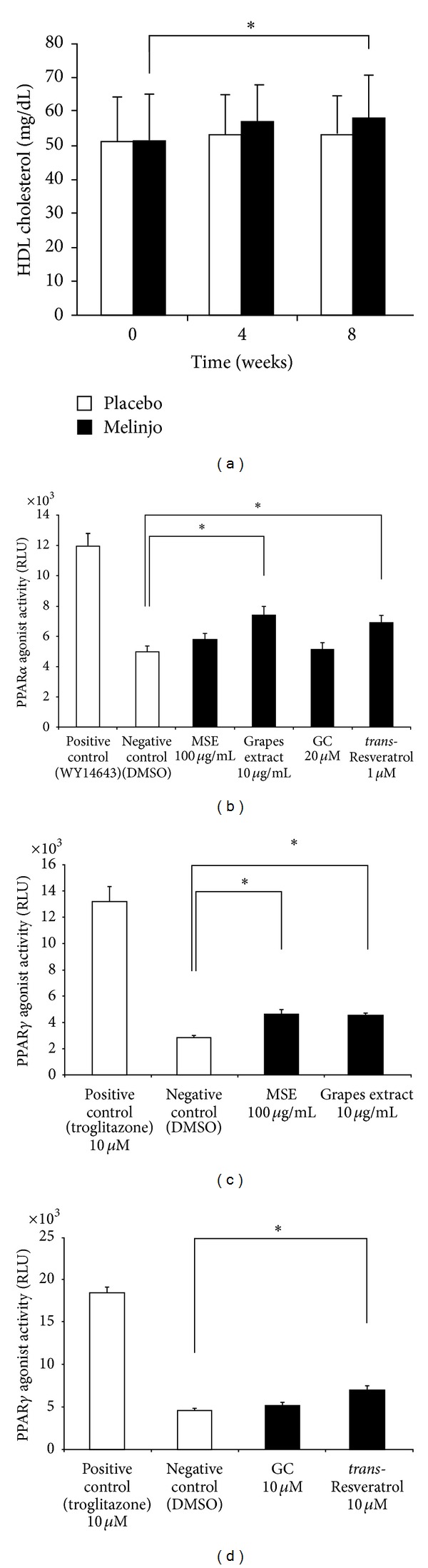
(a) The effects of MSE on the HDL cholesterol levels before and four or eight weeks after the administration of 750 mg MSE powder or placebo. Statistical analysis is presented in Figure 1. *P < 0.05. (b) The PPAR*α* agonist activity of MSE, grapes extract, GC, and *trans*-resveratrol. (c) The PPAR*γ* agonist activity of MSE and grapes extract. (d) The PPAR*γ* agonist activity of GC and *trans*-resveratrol. Values are presented as means ± SD. *P < 0.05.

Although we failed to detect any beneficial effects of MSE on LDL cholesterol, we found a significant increase in the HDL cholesterol levels in healthy volunteers who consumed 750 mg MSE powder for eight weeks as indicated in Figure 3(a) (52.4 ± 11.4 to 57.4 ± 12.6 mg/dL, *P* < 0.05). It is well known that HDL cholesterol transports the deposited cholesterol from atherosclerotic lesions of blood vessels to the liver [11], and it also counteracts the deleterious effect of LDL cholesterol in the pathogenesis of atherosclerosis. In this context, MSE may confer a benefit in the prevention of atherosclerosis without the alternation of LDL metabolism.

In order to clarify the molecular mechanisms that increase the HDL cholesterol levels on MSE administration, we evaluated the agonistic activities for PPAR*α* and PPAR*γ* because these receptors can increase the HDL cholesterol levels [12–15]. As displayed in Figures 3(b)–3(d), MSE or grapes extract revealed mild agonistic activities for PPAR*α* and PPAR*γ* (Figures 3(b) and 3(c)), which is similarly identified with *trans*-resveratrol (Figure 3(d)). The agonistic activities detected here revealed weaker signals compared with the positive controls, WY1643 for PPAR*α* and troglitazone for PPAR*γ* (Figures 3(b), 3(c), and 3(d)). For a better understanding of the mechanisms, further studies are warranted on the molecular mechanism involved in the metabolism of HDL cholesterol.

## 4. Discussion

The results of this study suggest that the MSE decreases the serum uric acid levels by inhibiting the reabsorption of uric acid in the renal tubular epithelia as well as by increasing the HDL cholesterol levels by PPAR agonistic activity. In this paper, we demonstrated, for the first time, the novel actions of MSE, which is distinct from *trans*-resveratrol. The actions demonstrated here have not been previously reported with *trans*-resveratrol [16–19].

In metabolic syndrome, insulin resistance causes hyperinsulinemia, which then leads to upregulation of serum uric acid by enhancing the reabsorption of serum uric acid in the renal tubules [20]. In addition, hyperinsulinemia downregulates GAPDH, one of the key glycolysis enzymes, which can then activate the pentose phosphorylation pathway with a concomitant increase of purine synthesis de novo [21]. It is unlikely that MSE downregulates uric acid by improving insulin resistance because we failed to identify any signs of improvement on HOMA-IR in the present study. This result suggests that MSE downregulates the serum uric acid levels independently of insulin resistance.

GC, gnemonoside C, and gnemonoside D in MSE are reported to inhibit the *α*-amylase activity [1]. This inhibitory activity could suppress the rapid postprandial insulin secretion, which inhibits the reabsorption of uric acid in the renal tubules. Notably, dysfunction of the ATP-binding cassette transporter subfamily G member 2 (ABCG2) suppresses the excretion of uric acid into intestine [22]. Another possibility is that MSE enhances ABCG2 to secrete more uric acid into the intestine, thereby decreasing the serum uric acid levels. Further clinical studies would be required to confirm the efficacy as well as the optimized MSE dose for an efficient control of uric acid and HDL cholesterol.

Regarding the influence of *trans*-resveratrol on uric acid, a few studies reported that in animals [23, 24]. In artificially induced hyperuricemia in mice, *trans*-resveratrol and its analogues decreased the serum uric acid levels and increased the uric acid excretion by regulating the renal organic ion transporters [23]. In addition, in diabetic rats, *trans*-resveratrol decreased the serum uric acid levels [24]. These findings suggest that *trans*-resveratrol decreases the serum uric acid levels in the presence of insulin resistance. Considering these results of studies, *trans*-resveratrol would not have influenced serum uric acid level in our study.

About the effects of *trans*-resveratrol on HDL cholesterol, some clinical trials have shown that *trans*-resveratrol increased HDL cholesterol. However, Sahebkar recently concluded that *trans*-resveratrol does not have a significant effect of resveratrol supplementation on plasma lipid concentrations in the meta-analysis of randomized controlled trials (RCT) [18]. In addition, the range of *trans*-resveratrol doses was between 10 mg/day and 1,500 mg/day in the RCTs [18], while the dose in our study was about 0.75 mg/day; it was very less than the selected studies. Therefore, *trans*-resveratrol would not have contributed to the increase of HDL cholesterol level in MSE group. However, it might be possible that *trans*-resveratrol was one of the ingredients in MSE that affected the parameters in our study since the effects of *trans-*resveratrol on serum uric acid and HDL-cholesterol still remain unclear.

## 5. Conclusions

Since the relation between hyperuricemia and metabolic syndrome has been pointed out these days [25], our study shed light on the possibility that MSE decreases the serum uric acid levels. Furthermore, MSE may improve the lipid metabolism by increasing the HDL cholesterol levels. Nevertheless, further research would be required to understand the molecular mechanism for regulating uric acid and HDL cholesterol, which is more specific for MSE and distinct from *trans*-resveratrol.

## Figures and Tables

**Table 1:** The effects of melinjo seed extract administration for 8 weeks in adult men.

	Week 0		Week 4		Week 8		*P*
	Melinjo (*n* = 14)	Placebo (*n* = 15)	Melinjo (*n* = 14)	Placebo (*n* = 15)	Melinjo (*n* = 14)	Placebo (*n* = 15)	
	Means ± SD	Means ± SD	Means ± SD	Means ± SD	Means ± SD	Means ± SD	
Body weight (kg)	70.8 ± 9.5	70.4 ± 5.9	71.5 ± 9.9	71.0 ± 6.4	71.4 ± 10.0	70.8 ± 6.2	0.96
Fat percentage (%)	22.1 ± 3.8	22.5 ± 3.4	22.9 ± 4.6	23.5 ± 3.2	22.9 ± 4.7	23.4 ± 3.4	0.946
BMI (kg/m^2^)	24.5 ± 2.6	24.3 ± 1.1	24.8 ± 2.6	24.5 ± 1.3	24.7 ± 2.6	24.4 ± 1.2	0.965
Systolic blood pressure (mmHg)	115.7 ± 10.7	118 ± 14.7	115.3 ± 9.2	118.9 ± 13.0	117.6 ± 10.5	119.5 ± 13.3	0.708
Diastolic blood pressure (mmHg)	74.9 ± 9.3	77 ± 11.8	75.7 ± 7.0	76.4 ± 10.4	75.2 ± 6.8	76.5 ± 9.3	0.756
Pulse (bpm)	60.6 ± 5.9	59.1 ± 8.7	61.3 ± 6.8	60.7 ± 8.6	62.9 ± 9.4	63.6 ± 11.8	0.711
FMD (%)	5.5 ± 2.6	5.8 ± 2.1	6.7 ± 1.6	6.1 ± 2.2	5.7 ± 2.4	6.8 ± 1.8	0.233
baPWV (right) (cm/s)	1210.4 ± 145.9	1297 ± 149.3	1236.5 ± 112.7	1282.5 ± 162.4	1283.5 ± 156.3	1314.9 ± 156.4	0.27
baPWV (left) (cm/s)	1226.1 ± 138.0	1324.1 ± 173.7	1241.9 ± 127.8	1305.1 ± 156.9	1299.7 ± 172.0	1337.5 ± 175.5	0.287
Ankle-brachial index (right)	1.14 ± 0.06	1.17 ± 0.06	1.13 ± 0.07	1.16 ± 0.04	1.13 ± 0.07	1.18 ± 0.04	0.549
Ankle-brachial index (left)	1.14 ± 0.05	1.16 ± 0.05	1.13 ± 0.07	1.14 ± 0.05	1.12 ± 0.08	1.16 ± 0.04	0.41
Total protein (g/dL)	7.1 ± 0.4	7.0 ± 0.3	7.12 ± 0.3	7.1 ± 0.3	7.2 ± 0.3	7.1 ± 0.4	0.156
Albumin (g/dL)	4.4 ± 0.2	4.4 ± 0.2	4.4 ± 0.2	4.4 ± 0.3	4.5 ± 0.2	4.4 ± 0.1	0.771
Total bilirubin (mg/dL)	1.0 ± 0.4	1.0 ± 0.7	0.9 ± 0.3	1.0 ± 0.8	0.9 ± 0.4	0.9 ± 0.7	0.956
Aspartate aminotransferase (U/L)	28.1 ± 10.6	21.7 ± 7.4	22.8 ± 5.6	22.1 ± 5.2	22.7 ± 5.9	22.0 ± 5.6	0.074
Alanine aminotransferase (U/L)	34.9 ± 24.8	25.4 ± 13.2	27.5 ± 15.2	27.8 ± 14.3	27.0 ± 14.1	26.7 ± 13.9	0.096
Gamma-glutamyl transpeptidase (U/L)	44.1 ± 33.8	36.3 ± 22.8	42.9 ± 35.0	42.9 ± 34.5	42.5 ± 40.7	42.1 ± 35.2	0.289
Total cholesterol (mg/dL)	193.9 ± 44.8	214.3 ± 26.7	196.4 ± 45.9	219.1 ± 28.1	199.0 ± 37.8	213.9 ± 29.7	0.654
HDL cholesterol (mg/dL)	52.4 ± 11.4	51.2 ± 12.9	54.1 ± 11.6	50.7 ± 11.4	57.4 ± 12.6	51.4 ± 13.7	0.111
LDL cholesterol (mg/dL)	122.9 ± 38.9	135.3 ± 27.8	123.5 ± 42.2	138.0 ± 24.3	124.5 ± 34.7	135.7 ± 30.0	0.871
Triglycerides (mg/dL)	106.3 ± 65.0	144.5 ± 104.7	114.7 ± 64.7	99.0 ± 47.7	118.5 ± 74.5	173.1 ± 136.6	0.381
Arteriosclerotic index	2.9 ± 1.4	3.4 ± 1.0	2.8 ± 1.2	3.5 ± 1.0	2.6 ± 1.1	3.4 ± 1.1	0.346
Uric acid (mg/dL)	6.7 ± 1.5	6.6 ± 1.1	6.3 ± 1.4	6.7 ± 0.9	6.1 ± 1.4	6.6 ± 1.1	0.009*
Blood urea nitrogen (mg/dL)	13.6 ± 3.7	13.8 ± 4.4	13.3 ± 4.0	13.3 ± 2.3	13.1 ± 3.7	12.9 ± 2.7	0.992
Creatinine (mg/dL)	0.9 ± 0.1	0.9 ± 0.1	0.8 ± 0.1	0.9 ± 0.1	0.8 ± 0.1	0.9 ± 0.1	0.283
Sodium (mEq/L)	139.9 ± 2.1	138.9 ± 1.8	138.9 ± 2.2	138.9 ± 2.6	139.1 ± 2.3	139.1 ± 2.4	0.629
Potassium (mEq/L)	4.3 ± 0.5	4.2 ± 0.3	4.4 ± 0.4	4.5 ± 0.3	4.4 ± 0.3	4.5 ± 0.4	0.284
Chlorine (mEq/L)	102.5 ± 2.5	102.2 ± 1.9	101.1 ± 2.3	101.9 ± 2.6	101.3 ± 2.2	101.9 ± 2.0	0.74
Blood sugar (mg/dL)	92.8 ± 13.7	92.6 ± 4.4	93.6 ± 13.5	93.5 ± 3.9	94.3 ± 18.7	95.9 ± 8.2	0.837
Hemoglobin A1c (%)	5.1 ± 0.5	5.0 ± 0.3	5.0 ± 0.7	5.0 ± 0.2	5.0 ± 0.7	5.1 ± 0.3	0.58
Albumin globulin ratio	1.7 ± 0.2	1.7 ± 0.2	1.6 ± 0.2	1.6 ± 0.3	1.6 ± 0.2	1.6 ± 0.2	0.666
White blood cell (×10^4^/*μ*L)	4846.7 ± 1186.2	6266.7 ± 2728.1	4760.0 ± 1025.3	6066.7 ± 2138.0	4860.0 ± 1240.3	6040.0 ± 2233.4	0.894
Red blood cell (×10^4^/*μ*L)	496.6 ± 31.6	480.1 ± 24.7	503.5 ± 34.6	491.1 ± 22.4	508.4 ± 28.3	483.3 ± 21.9	0.185
Hemoglobin (g/dL)	15.2 ± 0.6	14.9 ± 0.8	15.5 ± 0.7	15.4 ± 0.7	15.7 ± 0.6	15.2 ± 0.7	0.161
Hematocrit (%)	44.2 ± 2.1	43.2 ± 1.9	44.9 ± 2.0	44.6 ± 2.0	45.5 ± 1.7	44.0 ± 1.9	0.15
Mean cell volume (fL)	89.1 ± 2.7	90.3 ± 3.2	89.5 ± 3.1	90.9 ± 3.2	89.7 ± 3.5	91.3 ± 2.9	0.862
Mean cell hemoglobin (pg)	30.6 ± 1.4	31.0 ± 1.4	30.8 ± 1.5	31.5 ± 1.1	30.8 ± 1.5	31.5 ± 1.1	0.2
Mean cell hemoglobin concentration (g/dL)	34.3 ± 1.0	34.4 ± 0.8	34.4 ± 1.0	34.6 ± 0.7	34.4 ± 1.0	34.6 ± 0.7	0.756
Platelets (×10^4^/*μ*L)	23.2 ± 4.8	21.8 ± 5.4	24.0 ± 4.1	22.4 ± 6.3	24.3 ± 4.2	23.5 ± 7.7	0.685
Fasting IRI (*μ*U/mL)	5.1 ± 2.9	4.9 ± 2.2	5.4 ± 3.4	7.0 ± 5.4	5.4 ± 3.5	7.1 ± 6.5	0.407
HOMA-IR	1.2 ± 0.8	1.1 ± 0.5	1.3 ± 0.8	1.6 ± 1.3	1.3 ± 0.9	1.7 ± 1.7	0.404
NT-proBNP (pg/mL)	76.5 ± 195.4	32.1 ± 20.0	26.0 ± 16.7	26.2 ± 22.2	20.3 ± 14.4	25.1 ± 23.1	0.505
RLP cholesterol (mg/dL)	6.9 ± 4.8	8.9 ± 8.4	7.6 ± 5.3	13.0 ± 11.9	6.9 ± 4.9	11.0 ± 11.6	0.242
Total homocysteine (mg/dL)	13.5 ± 5.2	14.6 ± 8.4	11.7 ± 2.5	14.7 ± 8.9	11.2 ± 2.8	12.5 ± 4.6	0.399
d-ROMs test (U.CARR)	330.8 ± 58.4	331.6 ± 55.2	338.0 ± 53.6	345.6 ± 52.2	356.5 ± 46.2	365.0 ± 60.5	0.781
BAP test (*μ*mol/L)	2585.3 ± 174.2	2471.5 ± 189.3	2341.1 ± 91.9	2277.0 ± 115.7	2441.9 ± 178.5	2329.4 ± 179.9	0.633
Urine PH	5.7 ± 0.9	5.7 ± 1.2	5.5 ± 0.5	6.2 ± 1.1	6.1 ± 0.9	5.6 ± 0.8	0.008*
Urine specific gravity	1.021 ± 0.006	1.019 ± 0.008	1.023 ± 0.004	1.019 ± 0.008	1.022 ± 0.004	1.018 ± 0.007	0.689

BAP: biological antioxidant potential; d-ROMS: reactive oxygen metabolites-derived compounds; LDL: low-density lipoprotein; NT-proBNP: amino-terminal probrain natriuretic peptide; RLP: remnant-like particles. Values are given as means ± SD. *P* for interaction. *P < 0.05.
